# Pharmacotherapy for attention deficit/hyperactivity disorder in youth with avoidant restrictive food intake disorder: a case series of patients prescribed stimulant medication in a partial hospitalization program for eating disorders

**DOI:** 10.1186/s40337-023-00954-1

**Published:** 2023-12-18

**Authors:** Daphna M. Finn, Jessie E. Menzel, Emily Gray, Terry Schwartz

**Affiliations:** 1 UCSD Eating Disorder Center for Treatment and Research, University of California, 4510 Executive Drive Suite 315, San Diego, CA 92121 USA; 2 Equip Health, Carlsbad, CA USA; 3 Ohana at the Community Hospital of the Monterey Peninsula, Monterey, CA USA

**Keywords:** Attention deficit hyperactivity disorder, Avoidant restrictive food intake disorder, Stimulant medication, Children, Case series

## Abstract

**Background:**

Appetite suppression and weight loss are established potential side effects of most medications for attention deficit/hyperactivity disorder (ADHD). These side effects may be especially problematic when using stimulants to treat ADHD in the context of a restrictive eating disorder, such as avoidant restrictive food intake disorder (ARFID), although these diagnoses are often comorbid in children. This paper presents a combined approach to treating ADHD comorbid with ARFID using stimulant medication and behavior management within a partial hospitalization program (PHP) and intensive outpatient program (IOP)for eating disorders. The aim of this paper is to determine if the continued or new use of stimulant medication allows for adequate weight restoration by reviewing a series of cases receiving the combined treatment.

**Case presentations:**

Consecutive patients with a historical or new diagnosis of ADHD when presenting for treatment for ARFID were included in this case series. This series included 10 patients (8 male, 2 female) who received pharmacotherapy using stimulants and behavior management interventions involving structured mealtimes and contingency management. All treatment occurred within the context of a PHP/IOP for childhood eating disorders. All youth were able to effectively continue on stimulant medication, show clinical benefit in core ADHD symptoms, and able to gradually restore weight. In all cases, stimulant medications were not discontinued, but in some cases, doses were optimized (increased or decreased), switched to a different stimulant, or augmented with non-ADHD medication, such as mirtazapine, to support the management of ADHD while concurrently assisting in weight gain as necessary for the treatment of ARFID. Only one patient was newly started on a stimulant medication; as this was near the end of her treatment stay, limited conclusions can be drawn from this case.

**Conclusions:**

These findings support the use of pharmacotherapy, including continuing stimulant medication, when combined with behavior management strategies as a potentially effective treatment approach for ADHD in youth with ARFID in the PHP/IOP setting. Future studies using more rigorous methodology, longer follow-up times, and within other treatment settings are needed.

## Background

Attention deficit/hyperactivity disorder (ADHD) is a common neuropsychiatric condition that can cause significant educational and social impairment, with a recent meta-analysis showing an overall pooled estimate of 7.2% prevalence in youth aged 18 and under [1]. Although pharmacotherapy is one of the primary effective interventions for youth exhibiting ADHD, medication has been associated with appetite suppression [2, 3] and weight loss [4]. While these side effects are manageable in most circumstances and not a leading cause for discontinuation, for children and adolescents who are underweight and struggling with restrictive eating, they can be more problematic [5].

A meta-analysis showed that those with eating disorders have higher rates of comorbidity with ADHD by an odds ratio of 2.57 [6]. Given the potential problems with medication management of ADHD within this population, guidance on the successful management of these comorbid conditions is extremely relevant for clinical practice and information in this area is lacking.

The overlap between avoidant/restrictive food intake disorder (ARFID) and ADHD is of particular interest, as this eating disorder has a younger age of onset, a higher proportion of males compared to other restrictive eating disorders [7, 8], and this overlap may occur in 15–40% of patients [9, 10]. Existing literature on the treatment of these comorbid conditions is scarce and includes a single case report of 2 young patients with ARFID and ADHD treated with stimulants. These case reports indicated that stimulant medications exacerbated restrictive eating behaviors and lead to weight loss [11]. Based on the potential serious consequences of stimulant medication use in restrictive eating disorders, the authors encouraged future study of the dual treatment of these conditions in order to optimize outcomes.

To that end, we present a larger case series detailing the use of psychopharmacologic medications to target ADHD symptoms in patients with co-occurring ARFID and ADHD within a partial hospital program (PHP) and intensive outpatient program (IOP) for eating disorders. A common approach in our program is to combine ADHD medications (including stimulants) with behavior management strategies to enhance motivation and shape eating behavior during mealtimes.

Prior to undertaking this retrospective chart review, we had informally observed that in addition to improvement in ADHD symptoms such as inattention, impulsivity, and hyperactivity with stimulants, there were often other useful treatment effects that helped promote weight restoration. These included improved meal compliance and pacing at meals (i.e., eating within a specific timeframe). Eating and completing a meal is a goal-directed task; with improvement in ADHD management, we observed an improved focus on eating at mealtimes and reduced distractibility and hyperactive behavior. The use of increased structure at mealtimes, both in program and at home, likely also improved patients’ ability to persist with eating.

Other observations included a positive response to contingency management procedures. While reinforcement in and of itself may have promoted eating behavior, this response may also be the result of improved engagement with motivational systems. Neurobiological theories of ADHD implicate deficits in motivation-arousal systems as core features of the disorder [12]. Thus it is possible that effective medication management may improve patients’ abilities to respond to contingency management procedures and persist in their efforts to earn reinforcers.

The primary aim of this retrospective chart review was to take a closer look at our outcomes to determine if stimulant medication is safe and helpful in currently or recently underweight patients with ADHD and ARFID who require a higher level of care. Overall, based on these previous informal observations, we hypothesized that stimulant medication combined with behavior therapy in the therapeutic and highly structured setting of an eating disorder PHP/IOP would allow for adequate healthy weight restoration.

## Case presentations

### Method

#### Participants

This case series considered 14 consecutive patients diagnosed with ARFID and ADHD who were admitted for treatment to a PHP for child eating disorders between 2015 and 2019 at the University of California at San Diego Eating Disorders Center for Treatment and Research. These patients were referred to treatment by a variety of sources, including their outpatient medical and psychiatric providers, children’s hospitals to which the patients were admitted for medical stabilization, and some were self-referred. Six patients had ARFID characterized by low appetite and selective eating, one had low appetite only, two had selective eating only, and one had a fear of aversive consequences (choking). Families provided written consent for their treatment course and outcomes to be included in quality improvement projects and research studies that may be published. Four patients were excluded from the series given that they were not treated with stimulant pharmacotherapy for ADHD; 1 patient had never been on ADHD medication, 1 had an increase in aggression and decreased caloric intake after a single dose of a stimulant medication one month before starting the treatment program and the medication was discontinued prior to admission, and 2 were treated with alpha agonists only. Thus, this study describes a series of 10 patients (8 male, 2 female) ranging in age from 7 to 14 years old (mean = 10.3 years). The mean length stay in PHP/IOP was 91.4 days. Two patients required a second admission, one 13 months later and one 3.5 months later due to weight stagnation and return of prior eating disorder behaviors.

#### Procedure

The diagnosis of ADHD was conferred for all participants in one of two ways. First, some youth entered the program with a pre-existing diagnosis that was confirmed through clinical interview by the program psychiatrist. In other cases, a new diagnosis of ADHD was assigned during treatment based on a combination of unstructured clinical interviews with parents, input from school teachers, behavioral observation by program therapists, and structured assessment measures, such as the Conners’ Rating Scales (CRS) [13] or the Conners’ Continuous Performance Task (CPT) [14].

Parents consented to pharmacotherapy, patients assented, and program psychiatrists prescribed medication. The primary prescribing clinician was EG (a child and adolescent psychiatrist), with TS (a general psychiatrist) also occasionally treating patients. Prescribers met with PHP patients weekly and IOP patients monthly, attended weekly treatment team meetings with therapists, dieticians, and nurses, and spoke regularly with parents to assess the child’s progress. Prescribing clinicians provided psychoeducation to families about the risks and benefits of as well as alternatives to ADHD medication, including no medication. The study was completed as a retrospective chart review based on clinical notes from program therapists and psychiatrists.

#### Analyses

Due to the small sample size and retrospective study design, no quantitative analyses were undertaken. The primary outcome noted was whether a patient was able to successfully gain weight into their individualized healthy goal weight range (as determined by program registered dietician) while taking stimulant medication for ADHD. This was an open trial and there was no control group with ARFID and ADHD that was specifically excluded from taking stimulant medication.

#### Description of behavioral treatment

All patients received treatment within the context of a PHP and subsequently a stepdown IOP for child eating disorders. All patients attended the PHP 6 h per day, 5 days per week and the IOP for 3 h a day, 3–5 days a week. The day program included therapeutic meals and snacks, ongoing medical monitoring, nutritional and psychiatric assessment and management, and group, family, and individual therapy. The primary treatment modalities used were cognitive-behavioral therapy for ARFID (CBT-AR) [15] and family-based therapy for eating disorders (FBT) [16]. In addition to pharmacotherapy, an individualized, behavior management approach was utilized to improve ADHD symptoms and increase nutrition completion and promote subsequent weight gain. The components of the individualized behavior plan included structured mealtimes (use of time limits and timer, clear expectations of nutrition requirements, assigned seating at a table, and posted mealtime rules). Staff at mealtimes were instructed to provide prompts and reminders to stay on task as needed. In addition to the structured mealtimes, contingency management procedures were used to increase nutrition completion. Reinforcement tailored to the individual was used in response to completion of solid foods and/or nutritional supplements. Reinforcement included any of the following: access to electronics, program or at home privileges, stickers, small prizes from a prize box, or tokens that could be exchanged for larger items/privileges. Reinforcers were selected based on input from the patient, parent, and a program therapist.

## Results

Table 1 describes the demographic and clinical characteristics of the patients and their response to ADHD pharmacotherapy combined with behavior management.

**Table 1:** ARFID/ADHD case series

Pt #a	Age/gender	Race and ethnicity	Diagnoses	Comorbidities (psychiatric and medical)	Duration of ARFID	Length of stay in PHP/IOP (days)	Initial meds and doses	Discharge meds and dose	Admit weight (lbs)/% IBW	Dis-charge weight (lbs)/% IBW	Response to ADHD meds
1	14, M	White	-ARFID, low hunger cues -ADHD, combined type	None	2 yrs prior	53	Methylphenidate CR (Concerta®) 54 mg (off 1 week prior to starting treatment)	Methylphenidate 5 mg TID; guanfacine ER 1 mg	76.0/77.9%	90.2/92.5%	Better able to focus in group therapy; no significant difference in hunger noted when restarted stimulants in program
2	13, M	White	-ARFID, somatic complaints, taste/texture aversion -ADHD, unspecified	-ASD -somatic symptom d/o -failure to thrive	Since infancy	1st admission: 82 2nd admission (13 months later): 109	1st admission: methylphenidate CR (Concerta®) 54 mg; olanzapine 2.5 mg 2nd admission: methylphenidate CR (Concerta®) 18 mg; olanzapine 2.5 mg	1st admission: methylphenidate CR (Concerta®) 36 mg; olanzapine 2.5 mg; mirtazapine 7.5 mg 2nd admission: methylphenidate CR (Concerta®) 18 mg; olanzapine 5 mg; mirtazapine 7.5 mg	65.0/78.3% 80.2/86.7%	81.1/97.7% 91.0/98.4%	In first admission, methylphenidate CR was decreased from 54 to 27 mg at intake, as had decreased appetite in the past with it. Methylphenidate CR increased to 36 mg after 1 month of treatment (after initial decrease) was associated with increased control of ADHD but reduced appetite; was still able to gain weight at higher dose Between admissions methylphenidate CR was decreased further; parents found that at higher doses appetite was suppressed, and at lower doses ADHD was severe and pt got too distracted to eat at times
3	7, M	White, Hispanic/Latino	-ARFID, somatic complaints; taste/texture aversion; low hunger cues -ADHD, combined type	-Failure to thrive	1 yr prior	110	Lisdexamfetamine (Vyvanse®) 20 mg	Lisdexamfetamine (Vyvanse®) 30 mg; clonidine 0.2 mg qhs; melatonin 5 mg qhs	51.8/76.7%	69.8/103.4%	ADHD better controlled/focus improved when increased lisdexamfetamine to 30 mg. Mirtazapine was used during half of treatment stay but stopped before discharge due to night eating episodes
4	8, F	White	-ARFID, fear of choking -ADHD, unspecified	None	1 month prior	53	Escitalopram 2.5 mg	Methylphenidate (Metadate CD®) 10 mg	50.4/96.9%*	52.2/100.4%	Methylphenidate added late in treatment course (during last 1–2 weeks), not enough time to fully assess for change in ADHD symptoms
5	10, M	White	-ARFID, taste/texture aversion; low hunger cues -ADHD, inattentive type	-Anxiety unspecified	Since infancy	88	Lisdexamfetamine (Vyvanse®) 30 mg; melatonin qhs (unknown dose)	Lisdexamfetamine (Vyvanse®) 30 mg; clonidine 0.1 mg qhs	56.8/81.1%	67.2/96.0%	Difficulty concentrating dramatically reduced when started lisdexamfetamine prior to program, was able to abandon plans for individualized educational plan (IEP); no noticeable adverse effects of clonidine or lisdexamfetamine
6	11, M	White	-ARFID, taste/texture aversion; low hunger cues -ADHD, inattentive	-Anxiety unspecified	Since toddlerhood	121	Methylphenidate ER (Quillivant XR®) 42.5 mg; mirtazapine 15 mg qhs	Methylphenidate ER (Quillivant XR®) 42.5 mg; mirtazapine 15 mg qhs	61.4/83.0%	79.2/107.0%	ADHD symptoms well-controlled and stable on admission medication (current regimen was superior to multiple prior medication trials)
7	11, F	White	-ARFID, taste/texture aversion; low hunger cues -ADHD, combined	-Anxiety unspecified	About 5 yrs prior	137	Methylphenidate 20 mg qam and 15 mg qnoon; guanfacine ER 1 mg BID	Dexmethylphenidate (Focalin®) 10 mg TID; mirtazapine 30 mg; guanfacine ER 2 mg BID	57.6/77.8%	81.8/110.5%	Parents report that selective eating and low appetite predated use of ADHD medications, but low appetite further reduced on methylphenidate. Showed reduced impulsive behaviors, improved focus/attention after switched to dexmethylphenidate and there were no noticeable side effects
8	8, M	White	-ARFID, taste/texture aversion; low hunger cues -ADHD, inattentive	-Anxiety unspecified	Since infancy	81	Amphetamine-dextroamphetamine XR (Adderall XR®) 10 mg qam	Amphetamine-dextroamphetamine XR (Adderall XR®) 10 mg qam, mirtazapine 45 mg	51.0/81.0%	65.6/104.1%	ADHD symptoms well-controlled and stable on admission medication (parents noted patient became "a model student" and his oppositional behaviors and aggression decreased when stimulant was started, but they did notice mealtimes became more difficult)
9	13, M	White	-ARFID, taste/texture aversion -ADHD, inattentive type	-ASD -intellectual disability	Lifelong, worsened as he got older	1st admission: 67 2nd admission (3.5 months later): 12	1st admission: guanfacine 1.5 mg qam and 1 mg qhs, methylphenidate CR (Concerta®) 54 mg qday, risperidone 1 mg BID, lamotrigine 200 mg qam and 300 mg qhs 2nd admission: no med changes	1st admission: guanfacine 1.5 mg qam and 1 mg qhs, amphetamine-dextroamphetamine XR (Adderall XR®) 30 mg qam, lamotrigine 200 mg qam and 300 mg qhs 2nd admission: no med changes	89/82.4% 107.8/93.7%	102.4/94.8% 111.6/97.0%	Improved hyperactivity and increased meal completion in program with initial increase to methylphenidate CR 72 mg; with switch to amphetamine-dextroamphetamine XR, hyperactivity, impulsivity and attention were further improved
10	8, M	White, Hispanic/Latino	-ARFID (somatic complaints, taste/texture aversion, low hunger cues) -ADHD, combined type	-Anxiety unspecified -asthma	Since age 3	122	Methylphenidate CR (Concerta®) 36 mg qam, methylphenidate 10 mg qam and qafternoon, clonidine ER 0.1 mg BID, mirtazapine 7.5 mg qhs	Methylphenidate CR (Concerta®) 36 mg qam, methylphenidate 10 mg qam and 15 mg qafternoon,, clonidine ER 0.2 mg qhs, mirtazapine 30 mg qhs, aripiprazole 2 mg daily	47.0/90.4%*	49.6/95%	ADHD medications were started on inpatient pediatrics unit while patient was medically stabilized; he had improved hyperactivity, distractibility, inattention, frustration tolerance and aggression with treatment of ADHD

*These two patients received inpatient treatment for their eating disorder just prior to admission to PHP, so they were no longer significantly underweight at the time they began our program

^a^Pt #: Patient number

All but one patient started the program already taking stimulants. Medications were changed or dose adjusted on a variety of timelines, anywhere from the first to the last weeks of treatment, depending on clinical status and concerns. Most patients had positive responses to stimulant medications prior to starting program with respect to ADHD behaviors but no improvement in food intake or mealtime behaviors, and multiple did have reduced appetite and intake.

Of the 10 patients treated with stimulant medications, 2 continued on their admitting ADHD regimen with no change, 2 continued at an increased dose of stimulant (1 of these also added an alpha agonist), 1 continued at a decreased dose, 3 switched agents (2 were ultimately on a higher dose equivalent of the new agent and 1 on a lower dose equivalent; one in each group also added or increased an alpha agonist), 1 added an alpha-agonist to a stimulant, and 1 patient started a stimulant for the first time. No patient stopped stimulant medication entirely.

Although 7 out of 10 patients with ARFID reported chronically low hunger cues at baseline, weight restoration was still achieved in this group. Families largely felt that the benefits of ADHD medication outweighed the side effect of appetite suppression. This was evidenced by their continued support of providing a stimulant medication to their child while also understanding risks, benefits, and alternatives.

In many cases, additional medications were added to target poor appetite and assist with weight restoration. The most common non-ADHD medication added was mirtazapine (in 4 patients). An additional 5th and 6th patient admitted to the program already taking mirtazapine and the medication was maintained during treatment. Of the 4 patients started on mirtazapine, 1 patient discontinued the medication prior to discharge due to the emergence of night eating. Other medication augmentation included starting a selective serotonin reuptake inhibitor (2 patients), an antipsychotic (1 patient), and melatonin (1 patient). An additional 3 patients discontinued a selective serotonin reuptake inhibitor, antipsychotic, and melatonin during treatment (1 patient each).

## Discussion and conclusions

Overall, our data offers preliminary evidence that patients with ARFID and ADHD may be effectively and safely treated with stimulant medication when used in concert with behavioral strategies within the structured and monitored framework of a PHP/IOP setting.

Some patients experienced a reduction in appetite upon initiating or increasing the dose of stimulant therapy. Although these medications were less likely to exacerbate low hunger cues in individuals who already had them at baseline, in patients with other ARFID subtypes (taste/texture aversion, somatic complaints, or fear of aversive consequences) an appetite decrease may be more problematic as hunger cues are a primary motivator to eat despite other obstacles. When patients experienced appetite decreases that interfered in eating, mirtazapine was used to augment stimulant medication to improve appetite or decrease functional abdominal complaints that were barriers to food intake [17].

Other case series and case reports have suggested that mirtazapine is a useful agent in the treatment of ARFID, as it promotes increased appetite and weight gain, decreases nausea and vomiting, and improves gastric emptying [18–20]. While mirtazapine is an off-label approach in children, in our experience it is well tolerated despite initial sedation. Of the 5 patients discharged on mirtazapine, 2 were on it prior to admission and 3 were started on it during PHP/IOP. They were ultimately on doses ranging from 7.5 to 45 mg (mean 25.5 mg) at the time of completion of the program. Therefore, augmentation of stimulant medication with mirtazapine in addition to behavioral contingency management plans may further offset the negative side effects of stimulant medications on appetite in patients with ARFID.

As 9 of the 10 patients were already on stimulants prior to starting the PHP, our case series provides limited guidance on the most helpful timeframe to start psychopharmacological interventions for ADHD in patients who are concurrently receiving treatment for ARFID. Our data simply suggest that successful weight restoration is possible during continued treatment with stimulants at higher levels of care.

While we do not have long-term follow-up of patients, two patients required readmission to the PHP/IOP due to weight stagnation and the resurgence of eating disorder behaviors. Notable differences between these patients and the others in our sample were the presence of increased comorbidities. They were the only participants in our sample with autism spectrum disorder (ASD), and patient #9 also had an intellectual disability. These types of conditions might necessitate a greater degree of caretaker supervision at lower levels of care, especially as patients eat more meals at home or in school settings. They also may pose added challenges in treatment engagement andlocating suitable outpatient providers, make typical contingency management strategies that are helpful in other children less effective, or increase the likelihood of reduced appetite or other side effects associated with stimulant medication.

There are several significant limitations to the current study. As this data was from a case series, it did not include a control group and was not a blinded treatment trial. It included only patients who presented to our treatment center and is not demographically representative of all patients with ARFID and ADHD (for example, all 10 of our patients were White and two were Hispanic/Latino). Furthermore, there was no standardization in the timing of when medications were added or changed and no standardization with respect to the timing of the addition of contingency management plans. Thus, we cannot separate out the effects of the two interventions. Additionally, many patients were on multiple medications and we are unable to control for the potential impact these other medications may have had on treatment course. Finally, as these patients were treated at a higher level of care due to severity, more studies are needed to provide follow up metrics on stability of improvement after PHP/IOP discharge and assess whether these results could generalize to typical outpatient care settings. Despite these limitations, our study does indicate that stimulant medications can be used in the successful management of comorbid ARFID and ADHD at a higher level of care without negative impact on weight gain.

This case series provides the first published evidence that treating comorbid ARFID and ADHD with stimulants may be safe and effective in a structured treatment setting. All patients in the trial were able to gradually restore their weight, in addition to maintaining gains or showing improvement in their ADHD symptoms. In some circumstances, the use of stimulant medications actually allowed the patient to be more engaged and successful in the treatment program for ARFID. We highly encourage further study in this area, particularly at lower levels of care and community settings where combined treatment for ADHD and ARFID is more typically provided.

## Data Availability

The data provided in this paper was gleaned from retrospective chart review of individual patients’ admission notes, progress notes, and discharge summaries and was placed in an Excel file that was reviewed by the treating clinicians for accuracy.
